# Positive Correlation Between Acetabular Anteversion and Abduction in Developmental Dysplasia of the Hip: A CT‐Based Morphological Study

**DOI:** 10.1111/os.70037

**Published:** 2025-04-09

**Authors:** Tian Gao, Yuehao Hu, Yu Shan, Zhengquan Xu, Mengning Yan, Huiwu Li, Yuanqing Mao, Zanjing Zhai

**Affiliations:** ^1^ Shanghai Key Laboratory of Orthopaedic Implants, Department of Orthopaedic Surgery Shanghai Ninth People's Hospital, Shanghai Jiao Tong University School of Medicine Shanghai China; ^2^ Suzhou Ninth People's Hospital, Department of Orthopedics Suzhou Ninth Hospital Affiliated to Soochow University Suzhou China; ^3^ Suzhou Municipal Hospital, Department of Orthopedics Suzhou Hospital Affiliated to Nanjing Medical University Suzhou China

**Keywords:** acetabular abduction, acetabular anteversion, combined anteversion, developmental dysplasia of the hip, total hip arthroplasty

## Abstract

**Objective:**

Developmental dysplasia of the hip (DDH) exhibits abnormalities in hip anatomy, so changes in the acetabular and femoral angles hold clinical relevance. This study aimed to investigate the correlations between acetabular anteversion (AAV) and acetabular abduction (AAB), as well as between combined anteversion and combined abduction in patients with DDH, attempting to give evidence for synchronous torsion of the proximal femur and acetabulum. This study also aimed to propose a new method for predicting acetabular anteversion and combined anteversion angles, respectively, based on acetabular abduction and combined abduction angles on conventional pelvic radiographs.

**Methods:**

This retrospective study included 202 patients (404 hips) with DDH who underwent THA at our institution from February 2013 to September 2021. Preoperative AAB/femoral neck‐shaft angle (FNA) was recorded via radiograph. AAV/femoral anteversion (FA) was recorded via computed tomography and radiography. To assess the correlations between the AAV and AAB and between combined anteversion (sum of AAV and FA) and combined abduction (sum of AAB and FNA), linear regressions and Pearson's coefficients were calculated.

**Results:**

All hips were categorized into five DDH subgroups according to the Crowe classification: 93 normal, 140 Crowe type I, 68 Crowe type II, 59 Crowe type III, and 44 Crowe type IV. Fairly positive correlations were observed between combined anteversion and combined abduction in normal (*r* = 0.509), type I (*r* = 0.637), type II (*r* = 0.423), and type III (*r* = 0.511) subgroups. AAV and AAB demonstrated a moderate positive correlation in the normal (*r* = 0.508), type I (*r* = 0.511), type II (*r* = 0.516), type III (*r* = 0.332), and type IV (*r* = 0.603) subgroups.

**Conclusions:**

The AAV and AAB, as well as combined anteversion and combined abduction, exhibited positive correlations in normal and Crowe type I–III hips, suggesting the torsion of the acetabulum and synchronous torsion of the acetabulum and proximal femur. These findings quantify synchronized twisting of the hip and offer the potential significant implications for the accuracy of preoperative planning in THA, especially in DDH patients.

## Graphical abstract

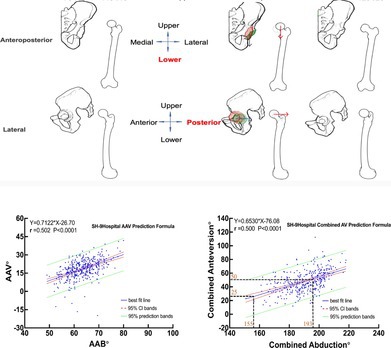

## 1 Introduction

Developmental dysplasia of the hip (DDH) presents as malalignment of the hip joint, causing aberrant stress and subluxation or dislocation [1, 2, 3, 4]. Total hip arthroplasty (THA) is a widely recognized treatment for DDH patients. Understanding hip development and morphometric characteristics in DDH patients is crucial for THA preoperative planning, including prosthesis selection and positioning to minimize postoperative complications.

**FIGURE 1:**
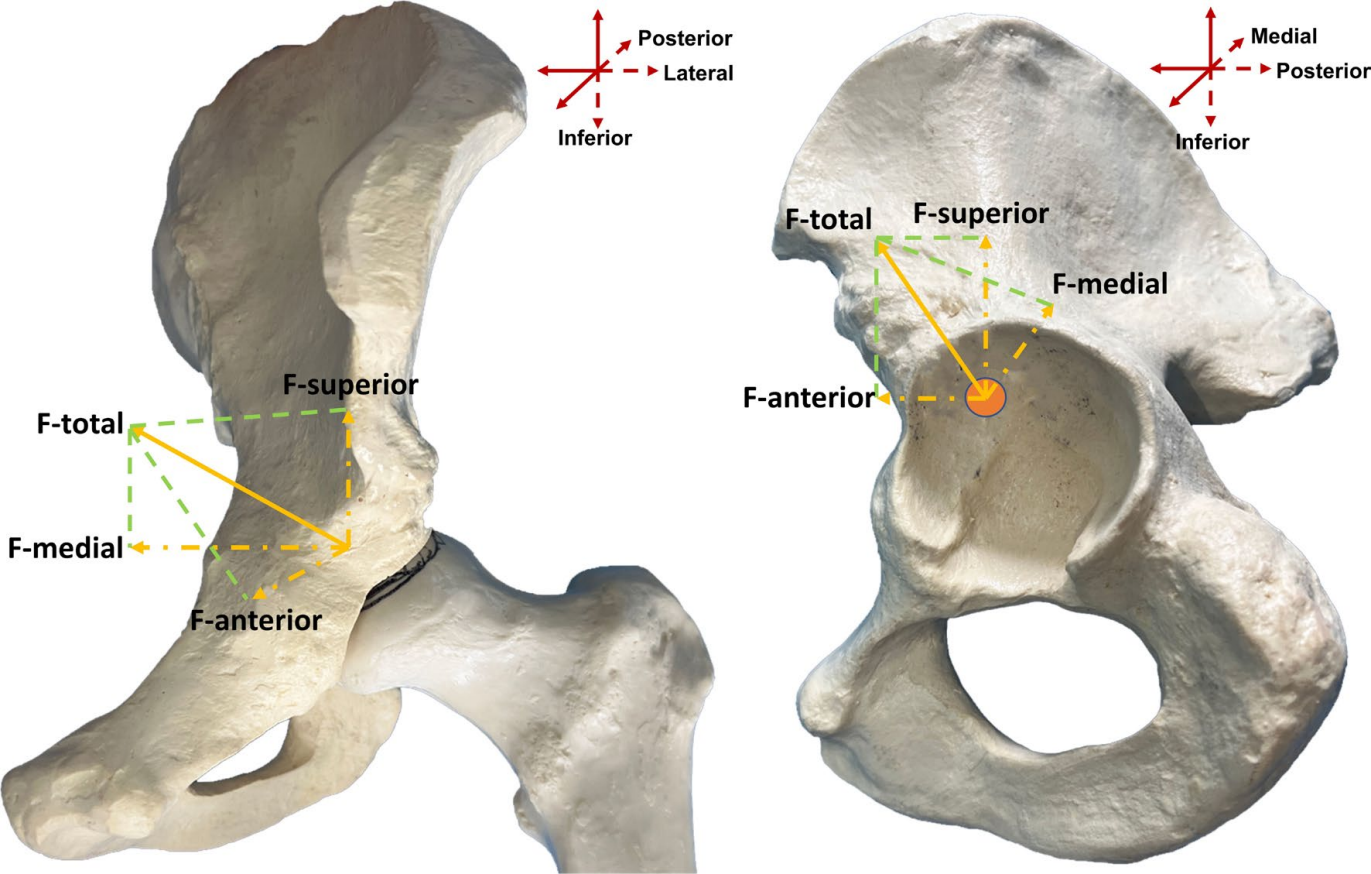
In vitro model showing the force direction and resolution for the acetabulum. (A) Coronal view of the force direction. (B) Sagittal view of the force direction.

**FIGURE 2:**
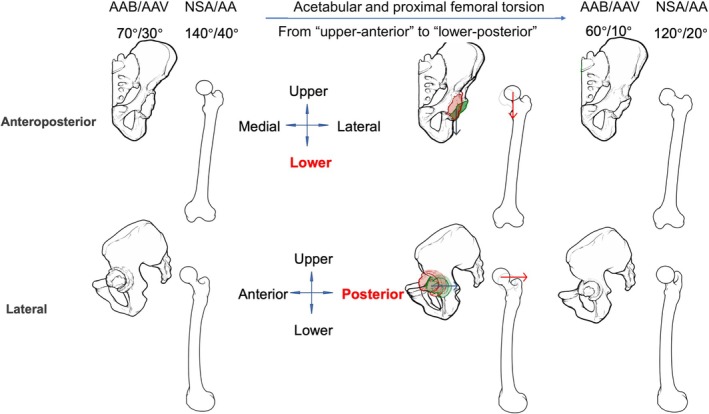
Diagram illustrating acetabular torsion.

Normal torsion of both the acetabulum and proximal femur is essential for hip congruency, which, in turn, is crucial for the proper development of both parts [5, 6]. Owing to abnormal biomechanical interactions between the acetabulum and proximal femur, patients with DDH exhibit abnormalities in hip anatomy, such as an increased acetabular anteversion (AAV) [7], increased femoral neck‐shaft angle (FNA), variations in the femoral anteversion (FA) [8]. Among these, changes in the acetabular and femoral angles hold clinical relevance. Okuzu et al. found that FNA is an influencing factor of acetabular morphology (acetabular width ratio) [9]. The concept of combined anteversion [10] (also known as the Mckibbin index), which is the sum of AAV and FA, has been previously proposed, which has been widely used for preoperative planning of the THA safe zone. Owing to the close relationship between development and force, we believe that the acetabulum and proximal femur should be treated as a whole, not only in the axial plane but also in the coronal plane. However, the corresponding definitions and numerical ranges have not yet been reported and remain unknown. On this basis, we proposed the concept of combined abduction, that is, the combination of acetabular abduction (AAB) and proximal femoral abduction. Since the degree of proximal femoral abduction in the coronal plane can be presented as the FNA (Figure 2), we proposed the combined abduction as the sum of AAB and FNA. Defining combined abduction based on the concept of combined anteversion could provide a three‐dimensional framework for optimizing prosthesis alignment, which is not yet standardized in current protocols. Variations in the acetabular and femoral anatomy angles are related to the precession of acetabular and femoral torsion during development. Furthermore, maltorsion leading to acetabular or proximal femoral abnormalities can result in joint instability, subluxation, or dislocation [11]. Additionally, the increasing recognition of variability in the patterns of acetabular and proximal femoral morphology has become crucial in the diagnosis and management of hip dysplasia [12]. The proximal femur experiences the influence of mechanical forces and twists downwards and backwards simultaneously, resulting in a significant correlation between proximal femoral anteversion and abduction [13, 14]. Moreover, Polkowski et al. proposed a model describing the conduction of mechanical forces in the lower extremities [15]. The hip joint serves as a pivot that regulates force transmission from the upper trunk to the lower extremities (Figure 1), with the simplified force static diagram (Figure 2) illustrating this direction as “inferior‐lateral‐posterior” to “superior‐medial‐anterior.” Nonetheless, deeper insights into the mechanisms underlying acetabular and femoral torsion were still limited.

Our previous work demonstrated a positive correlation between FNA and FA [16]. Previous studies have clarified that using combined anteversion alone as a prosthesis placement indicator is difficult to prevent dislocation after THA [17]. Therefore, this study aimed to (i) demonstrate whether the acetabulum and proximal femur undergo synchronous torsion in a three‐dimensional orientation during development due to interactional force; (ii) propose for the first time the concept of combined abduction (the sum of FNA and AAB) as a coronal supplementary reference for preoperative planning and further analyze the correlation between combined abduction and combined anteversion.

## 2 Materials and Methods

### 2.1 Study Participants

The study was conducted in accordance with the ethical principles embodied in the Declaration of Helsinki and its later amendments and was approved by the Medical Ethics Committee of Shanghai Ninth People's Hospital, Shanghai Jiao Tong University School of Medicine (SH9H2021‐T238‐2). The requirement for the acquisition of informed consent from patients was waived due to the retrospective nature of this study.

This retrospective study was implemented at our institution and included patients with DDH who underwent THA at our institution from February 2013 to September 2021. The inclusion criteria were as follows: patients who [1] were 18–85 years of age; [2] were diagnosed with either unilateral or bilateral DDH according to the Crowe classification; and [3] had available preoperative computed tomography (CT) images and radiographs of the pelvis and lower extremities for the measurement of the FNA/FA and AAV/AAB. The exclusion criteria were as follows: [1] previous hip surgery, such as osteotomy, or conservative orthopedic treatment; [2] obvious hip flexion contracture, secondary OA with osteophytes or femoral neck fracture [18]. The bilateral hips of all included patients were classified into Crowe types I–IV according to the Crowe classification, unless they were normal.

### 2.2 Radiographic and CT Assessment

**FIGURE 3:**
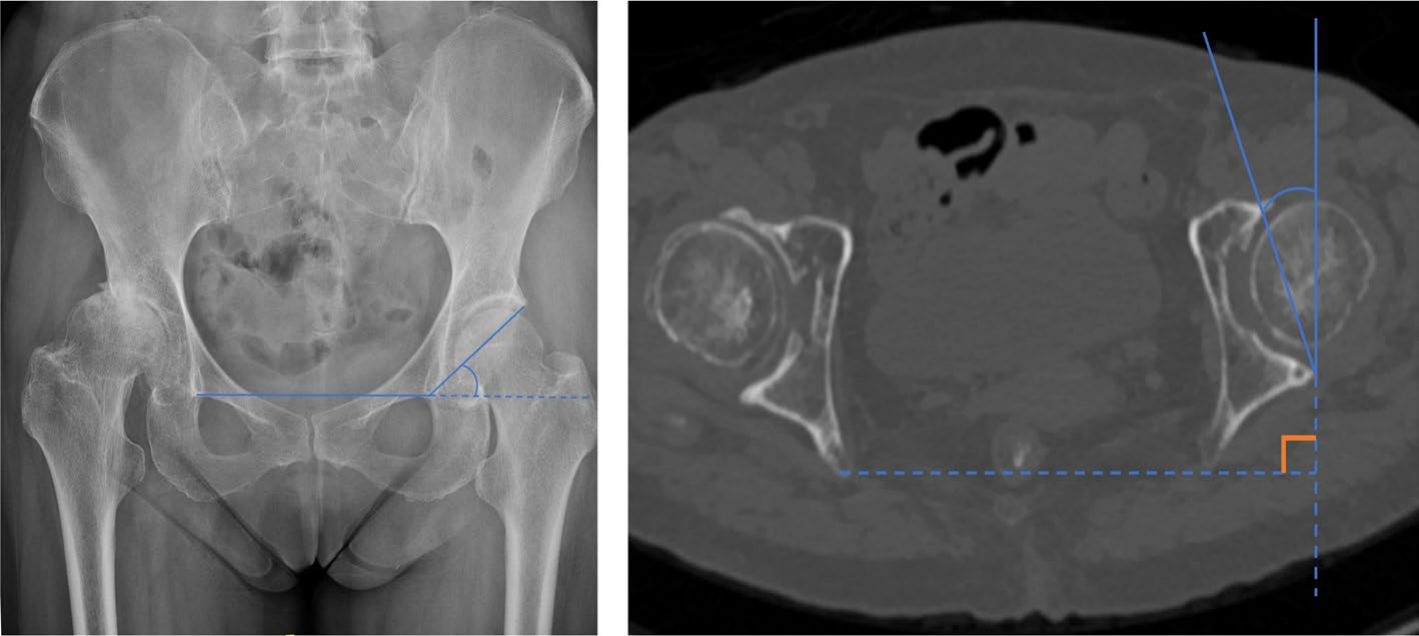
Illustrations showing the anatomical parameters of the acetabulum. (A) Acetabular abduction angle measurement. (B) Acetabular anteversion angle measurement.

Anteroposterior pelvic radiographs were obtained with the X‐ray beam centred on the superior aspect of the pubic symphysis and perpendicular to the patients. The AAB, a radiographic parameter that reflects the coverage of the acetabulum to the femoral head, was measured by determining the angle between a line drawn from the acetabular teardrop to the lateral acetabular margin and a horizontal line between the teardrops [16, 19] (Figure 3A). The FNA is determined by the medial angle formed between the femoral neck axis and the femoral shaft axis in plain radiographs (Figure S1).

With a GE ProSpeed CT scanner (GE HealthCare, Chicago, IL, USA), CT images were obtained at 2‐mm intervals from the anterior superior iliac spines to the inferior rim of the pelvis, covering the bilateral anterior iliac spine and posterior borders of the medial and lateral condyles. On axial CT images at the level of the mid‐femoral head, the AAV was measured by determining the angle formed by the intersection of a line drawn tangent to the anterior and posterior acetabular edges and a line perpendicular to the anterior pelvic plane in the axial plane [20] (Figure 3B). Based on three‐dimensional reconstructions, FA was defined on the axial plane as the angle between the femoral neck axis and the posterior condylar line (Figure S2), as described in detail in our previous article.

Two senior orthopedic physicians independently performed all angle measurements twice, with an interval of > 2 weeks between measurements. The average angle values were used for statistical analyses.

### 2.3 Statistical Analyses

Statistical analyses were performed via SPSS version 26.0 (IBM Corp., Armonk, NY, USA) and Prism version 9.3 (GraphPad Software Inc., San Diego, CA, USA). Descriptive statistics, including means, ranges, and standard deviations, were calculated to describe the AAV and AAB distributions. Considering the large sample size, the normality of the variables' distributions was examined via the Kolmogorov–Smirnov test. Angle values were compared among different subgroups via one‐way analysis of variance, whereas further pairwise comparisons between subgroups were conducted via Dunnett's *t‐*test. Intraclass correlation coefficients (ICCs) were calculated to evaluate the interobserver and intraobserver reliability. Correlations between combined anteversion and abduction and between AAV and AAB were assessed via Pearson's correlation coefficients, with the strength of the correlation being interpreted as follows: *r* = 0.00–0.19, negligible; *r* = 0.20–0.39, weak; *r* = 0.40–0.59, moderate; *r* = 0.60–0.79, strong; and *r* = 0.80–1.00, very strong. The relationships between AAB and AAV and between combined anteversion and combined abduction in each subgroup were evaluated via linear regression models. Additionally, a 95% confidence interval (CI) was applied, and statistical significance was set at a *p* value of < 0.05.

## 3 Results

### 3.1 Demographics

A total of 202 patients diagnosed with DDH, who subsequently underwent THA at our institution between February 2013 and September 2021, were included in this study. This cohort contained 103 male and 99 female patients, with a mean age of 58.4 ± 16.6 years and a mean body mass index (BMI) of 23.4 ± 5.0 kg/m^2^. In total, 404 hips were analyzed, consisting of 202 left and 202 right hips. The 404 bilateral hips were classified into five subgroups according to the Crowe classification: normal (*n* = 93), Crowe type I (*n* = 140), Crowe type II (*n* = 68), Crowe type III (*n* = 59), and Crowe type IV (*n* = 44).

### 3.2 Intraobserver and Interobserver Reliability

The ICCs for the intraobserver reliability of the AAB and AAV were 0.87 (95% confidence interval [95% CI]: 0.84–0.90, *p* < 0.001) and 0.85 (95% CI: 0.82–0.89, *p* < 0.001), respectively, whereas the ICCs for the interobserver reliability of the AAB and AAV were 0.88 (95% CI: 0.84–0.91, *p* < 0.001) and 0.86 (95% CI: 0.82–0.90, *p* < 0.001), respectively.

### 3.3 Measurements of AAV, AAB, Combined Anteversion, and Combined Abduction

**FIGURE 4:**
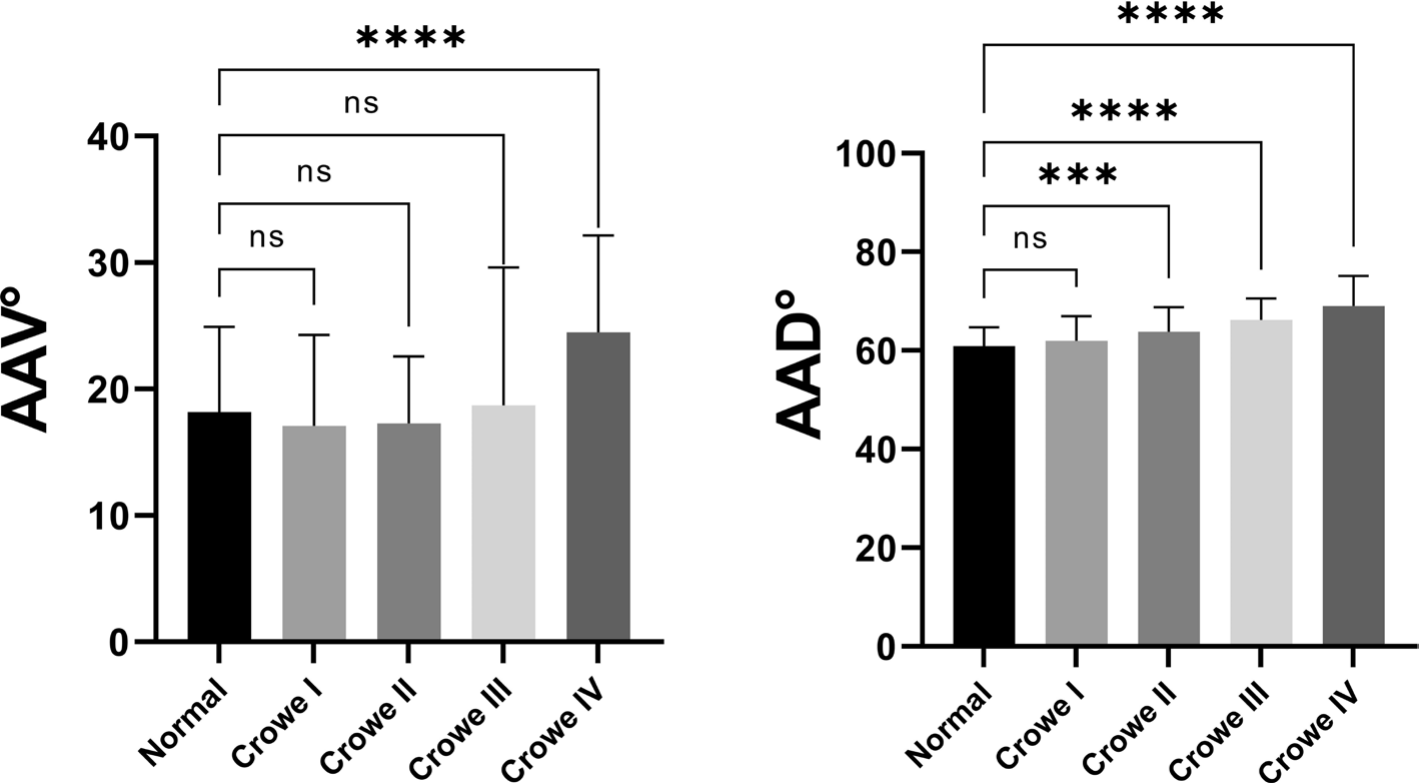
Angle values measured in five patient subgroups for the AAV (A) and AAB (B).

**FIGURE 5:**
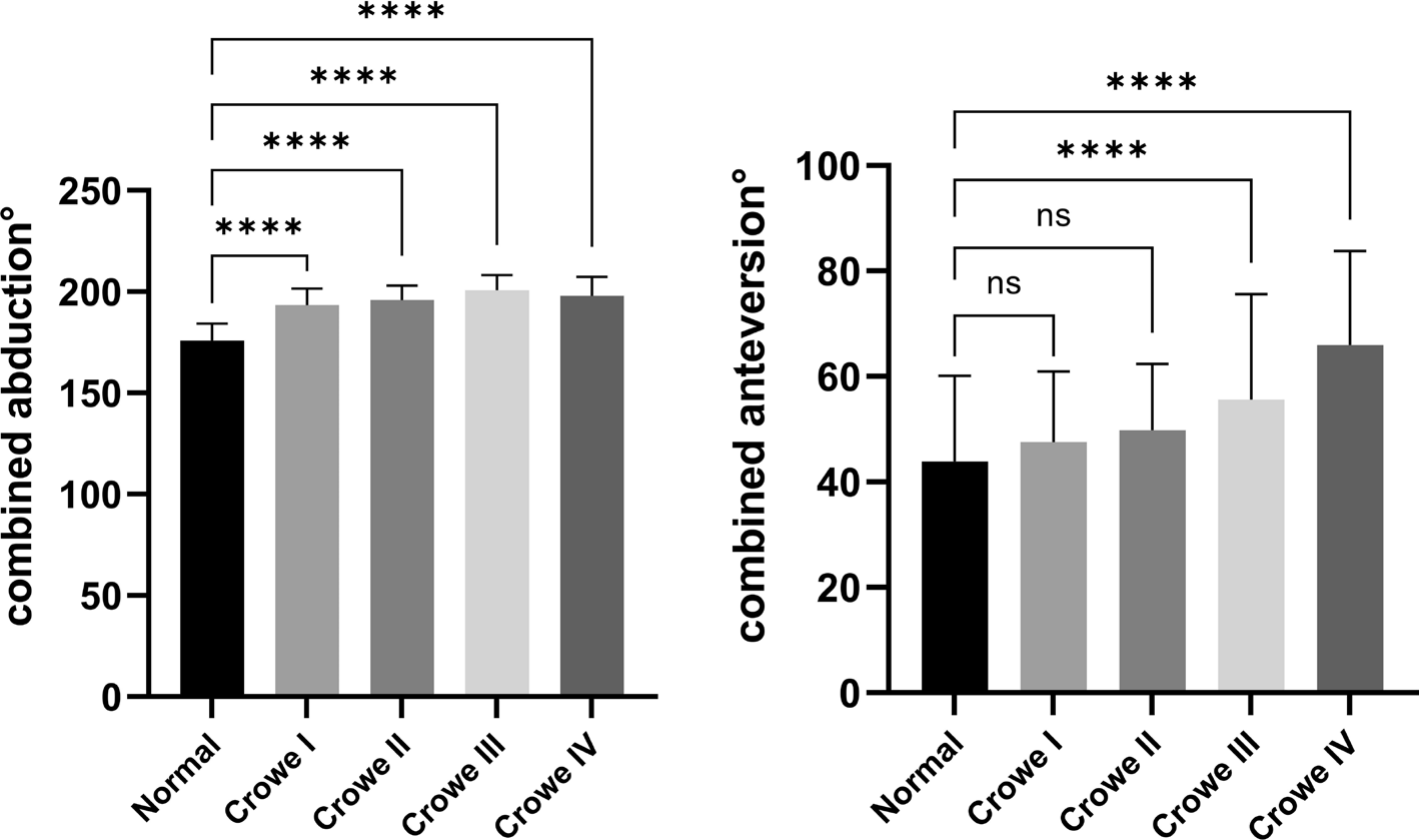
Angle values measured in five patient subgroups for combined anteversion (A) and combined abduction (B).

Among all 404 hips, the average AAV was 18.4° ± 6.2° in the normal subgroup and 17.0° ± 7.2°, 17.3° ± 5.3°, 18.7° ± 10.8°, and 24.5° ± 7.6° in the Crowe type I–IV subgroups, respectively (Table 1). On the basis of a previous study [16], we further calculated the combined anteversion and combined abduction angle. The average combined anteversion angle was 43.9° ± 16.2° in the normal subgroup and 47.5° ± 13.4°, 49.8° ± 12.5°, 55.6° ± 19.8°, and 65.9° ± 17.7° in the Crowe type I–IV subgroups, respectively. Further analysis revealed no significant difference in AAV values between the Crowe type I–III subgroups and the normal subgroup (*p* > 0.05); however, hips in the Crowe type II–IV subgroups had significantly greater AAB values than those in the normal subgroup (*p* < 0.001) (Figure 4). With respect to the combined anteversion, the analysis revealed a greater degree of combined anteversion in the Crowe type III and IV subgroups (*p* < 0.001) (Figure 5).

**TABLE 1:** Dimensional parameters of all the hips and subgroups of hips.

Parameters	Overall (*N* = 404)	Normal (*N* = 93)	Type I (*N* = 140)	Type II (*N* = 68)	Type III (*N* = 59)	Type IV (*N* = 44)
AAV (°)	18.4 ± 7.7	18.4 ± 6.2	17.0 ± 7.2	17.3 ± 5.3	18.7 ± 10.8	24.5 ± 7.6
AAB (°)	63.4 ± 5.4	60.9 ± 3.7	62.0 ± 5.0	63.8 ± 5.0	66.2 ± 4.4	69.0 ± 5.4
Combined AV (°)	50.3 ± 16.8	43.9 ± 16.2	47.5 ± 13.4	49.8 ± 12.5	55.6 ± 19.8	65.9 ± 17.7
Combined ab (°)	191.3 ± 12.0	175.8 ± 8.4	193.4 ± 8.2	195.9 ± 7.2	200.7 ± 7.5	198.0 ± 9.4

*Note*: Data are presented as mean ± standard deviation. All values are mean ± standard deviation.

Abbreviations: AAB: acetabular abduction; AAV: acetabular anteversion; AB: abduction; AV: anteversion.

### 3.4 Pearson Correlation Coefficient and Linear Regression Analysis of AAV and AAB, Combined Anteversion, and Combined Abduction

**FIGURE 6:**
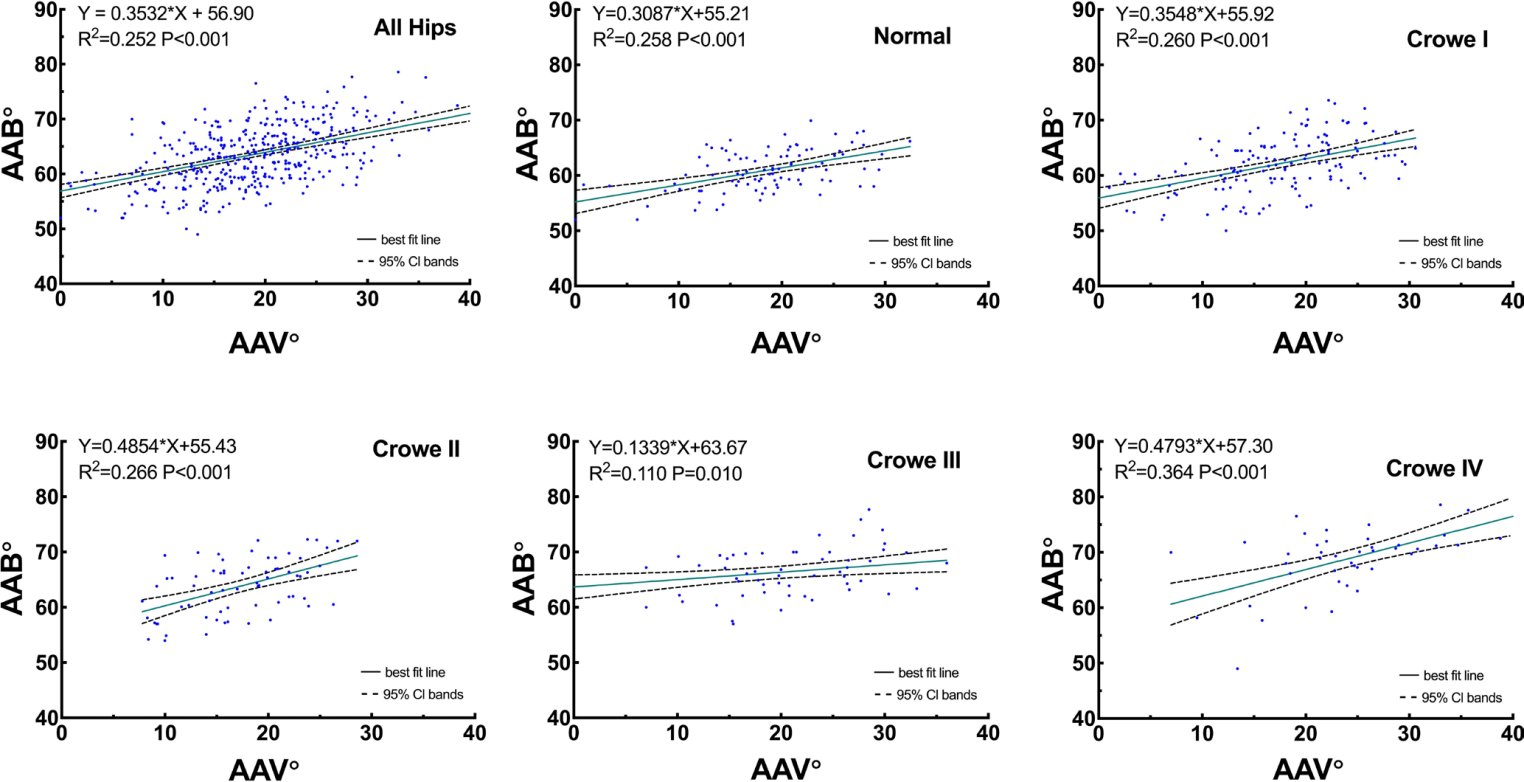
Correlation analysis between the AAV and AAB. Correlation analysis of (A) all hips, (B) normal hips, (C) Crowe I hips, (D) Crowe II hips, (E) Crowe III hips, and (F) Crowe IV hips.

**FIGURE 7:**
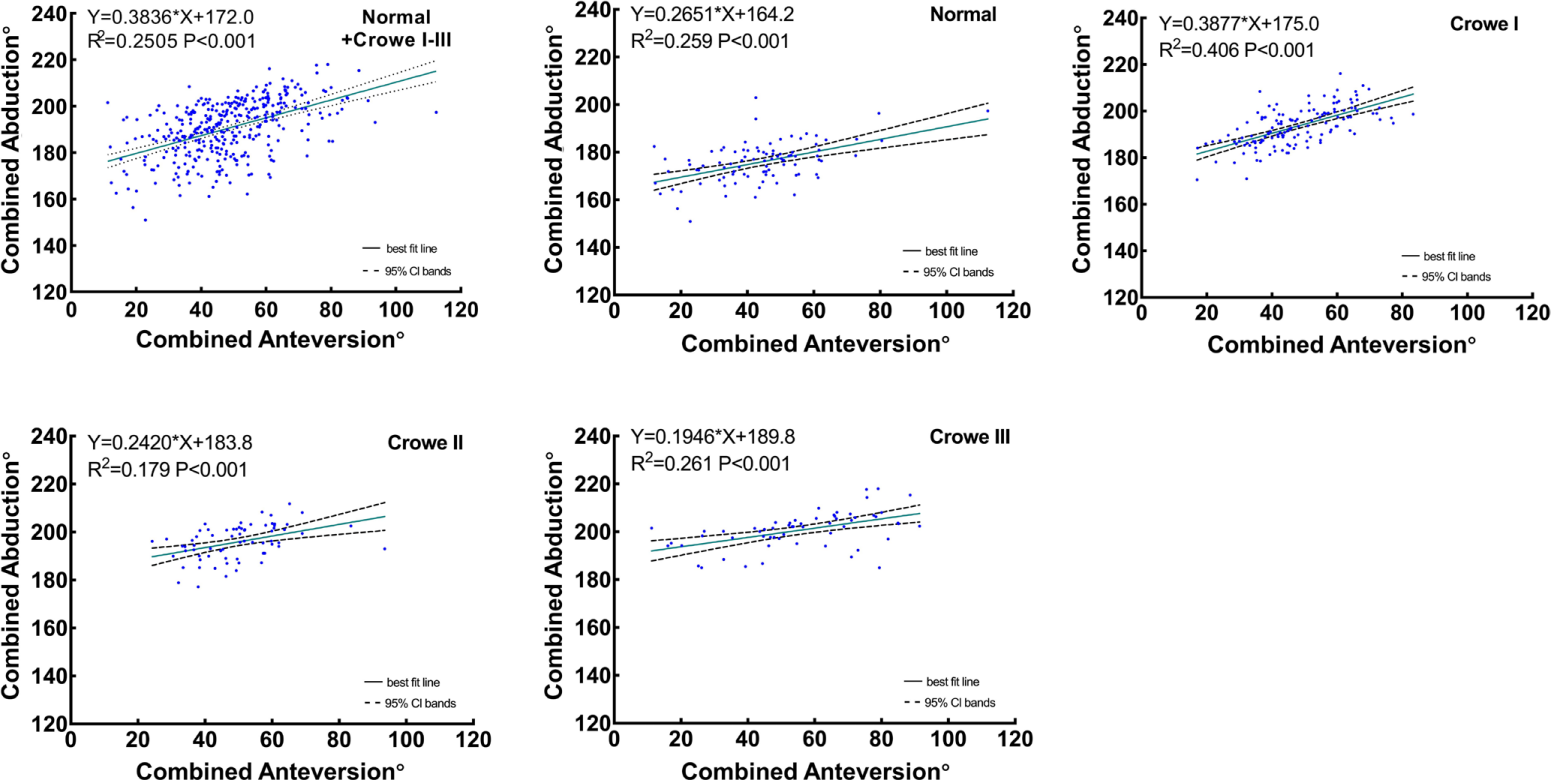
Correlation analysis between combined anteversion and combined abduction. Correlation analysis of (A) normal and Crowe I–III hips, (B) normal hips, (C) Crowe I hips, (D) Crowe II hips, and (E) Crowe III hips.

**FIGURE 8:**
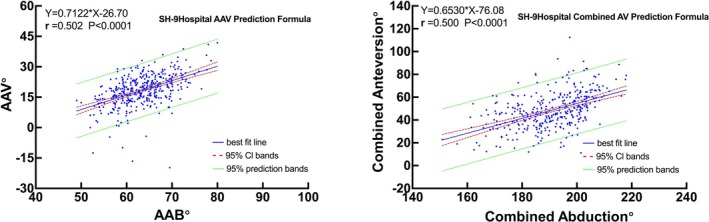
Linear regression analysis for predicting AAB based on AAV measurements of normal and Crowe type I–IV hips (A) and for predicting combined abduction based on combined anteversion measurements of normal and Crowe type I–III hips via the SH‐9 Hospital Prediction Formula (B).

We calculated Pearson's correlation coefficients and performed a linear regression analysis with the AAV and AAB as the independent and dependent variables, respectively. Pearson's correlation analysis of all the hips revealed a moderately positive correlation between the AAV and AAB (*r* = 0.502, *p* < 0.001). The AAV was moderately correlated with the AAB in the normal (*r* = 0.508, *p* < 0.001), Crowe type I (*r* = 0.510, *p* < 0.001), Crowe type II (*r* = 0.516, *p* < 0.001), and Crowe type IV (*r* = 0.603, *p* < 0.001) subgroups. Contrarily, the AAB was weakly correlated with AAV in the Crowe type III subgroup (*r* = 0.332, *p* = 0.010) (Figure 6). The same calculations were applied to the combined angles, with the combined anteversion as the independent variable and the combined abduction as the dependent variable. Pearson's correlation analysis revealed a moderately positive correlation between the combined anteversion and combined abduction in normal and Crowe I–III hips (*r* = 0.500, *p* < 0.001), establishing an original prediction formula as combined anteversion° = 0.6530 × combined abduction − 76.08 (Figure 8, SH‐9Hospitol Combined AV Prediction Formula) for calculating the combined anteversion value. The combined anteversion showed a moderate to strong correlation with the combined abduction in the normal (*r* = 0.509, *p* < 0.001), Crowe type I (r = 0.637, *p* < 0.001), Crowe type II (*r* = 0.423, *p* < 0.001), and Crowe type III (*r* = 0.511, *p* < 0.001) subgroups (Figure 7). Since there is no actual contact surface between the true acetabulum and the femoral head in Crowe type IV as the false acetabulum contacts the femoral head instead, the correlation between the combined angles of Crowe type IV was not discussed.

## 4 Discussion

In this study, we analyzed the radiographic data of 404 normal and dysplastic hips, and the results revealed a moderately positive correlation between AAV and AAB, and similarly between combined anteversion and combined abduction. These results are consistent with previous studies that suggest the simultaneous and synchronized twisting of the acetabulum and femur under mechanical forces.

Furthermore, based on the results, we proposed a novel and practical method for estimating AAV using AAB and predicting combined anteversion through combined abduction (Figure 8), using radiography data. Furthermore, based on the generally accepted safe zone of combined anteversion of 25°–50° [21, 22], with the SH‐9Hospitol Combined AV Prediction Formula (Figure 8), we consider the corresponding safe zone of combined abduction is 155°–193°.

### 4.1 Correlation Between AAV and AAB and Between Combined Anteversion and Combined Abduction Reflects the Synchronous Torsion of the Hip

The clinical significance of this study lies in its potential to enhance understanding of hip joint mechanics, particularly in patients with DDH. DDH has been recognized as a risk factor for early‐onset osteoarthritis [15]. Previous studies have shown that even less severe acetabular deformities contribute to early joint degeneration [4, 16, 23, 24]. Clohisy et al. [25] examined a cohort of 710 hips treated with THA and reported that DDH and femoral acetabular impingement accounted for premature OA in 48.4% of hips, whereas 9.5% and 6.2%, respectively, presented evidence of Legg‐Calvé‐Perthes disease or slipped capital femoral epiphysis. Similarly, Wyles et al. [26] reviewed the contralateral hips of 722 patients who underwent THA and reported that patients with DDH presented a greater risk of progressing to advanced stages or requiring THA. Therefore, understanding the process of hip torsion and measuring dysplastic hips during development could be promising for identifying potential strategies to improve hip congruence and morphology. The results of this study may be attributed to the fact that continuous stress on the growing hip can lead to morphological adaptations that simultaneously decrease the combined anteversion and combined abduction parameters.

Normal development of the acetabulum, which occurs at the level of the triradiate cartilage and acetabular cartilage [27], depends on the presence of a normally growing and functioning femoral head [28]. The evidence also suggests that external factors can modify growth at a physical level [29]. Considering the results of our previous research, the present study assessed the correlation between AAV and AAB, as well as between combined anteversion and combined abduction. The correlation between the combined angles and between the femoral angles was slightly stronger than that of the acetabulum.

The AAB was positively correlated with the AAV, illustrating that the acetabulum twists backwards and downwards under mechanical stimulation during development, which is three‐dimensional and synchronous. This finding is consistent with the results of our previous research on the mechanism underlying proximal femoral torsion [16]. The combined abduction and combined anteversion showed a stronger correlation, further verifying our hypothesis that torsion of the acetabulum and proximal femur is closely related and synchronous due to interacting forces. Factors including body weight, gait, exercise mode (e.g., golfing) [30], and exercise volume during growth may affect the development of the skeleton, which has an elastic modulus, and may influence the mechanics of the local hip joint, indicating potential directions for future research.

### 4.2 Clinical Implications and Research Potential

Our findings highlight a positive correlation between combined anteversion and combined abduction, indirectly illustrating synchronized twisting of the acetabulum and proximal femur. These findings support the concept of combined abduction and its application in THA. In THA, ensuring hip joint stability is essential and depends on the relative position of the prosthesis and acetabular bone coverage. On the basis of combined anteversion, we present the concept of combined abduction. Clinically, the FNA and AAB could be easily measured via pelvic X‐ray, allowing the use of the “SH‐9 Hospital Combined Anteversion prediction Formula” to estimate the combined anteversion value without CT scans. Given that adjusting femoral prosthesis anteversion is limited, a large natural combined abduction may reduce anterior coverage, causing femoral or iliopsoas impingement, whereas a small combined abduction may reduce posterior coverage, leading to other complications. Thus, surgeons generally modify combined anteversion to reduce the risk of instability, impingement or compromise long‐term outcomes. These insights are expected to help anticipate the challenges of preoperative planning and intraoperative prosthesis placement in THA.

### 4.3 Limitations and Strengths

In this study, we verified the correlation between AAV and AAB, as well as synchronous torsion of the hip based on the large sample of DDH patients, and further presented a novel and simple method for predicting AAV/combined anteversion by using AAB/combined abduction from radiographs. However, our study has several limitations. The data were obtained from Chinese adults at our institution; the association between AAV and AAB may differ between races and nationalities [31], since different countries and races have different lifestyles and exercise habits. Furthermore, during pelvic CT scanning, the assumed uniform position of patients may vary depending on their size, presence of spine deformities, and hip/lower extremity contractures, which can alter acetabular measurements. Moreover, the force static diagram we used in our analysis was a simplification of in vivo exerted forces, whereas the actual situation is a more complex mechanical process.

### 4.4 Conclusions

There is a significant correlation between combined anteversion and abduction in normal and Crowe type I–III hips, underscoring the importance of treating acetabular and femoral angles as a whole in hip development. The linear relationship between the AAB and AAV provides a reference for estimating the AAV values based on the AAB values.

## Author Contributions

All authors had full access to the data in the study and take responsibility for the integrity of the data and the accuracy of the data analysis. Conceptualization, T.G. and Y.H.; Methodology, T.G. and Y.S.; Investigation, T.G., Y.H., Z.X., and M.Y.; Formal Analysis, T.G. and Y.H.; Resources, H.L. and Y.M.; Writing – Original Draft, T.G., Y.H., and Z.Z.; Writing – Review and Editing, T.G., Y.H., and Z.Z.; Visualization, Y.H.; Supervision, Z.Z. and Y.M.; Funding Acquisition, Z.Z. and Y.H.

## Disclosure

The authors have nothing to report.

## Ethics Statement

This study was conducted according to the guidelines of the Declaration of Helsinki and approved by the Institutional Review Board of Shanghai Ninth People's Hospital, Shanghai Jiao Tong University School of Medicine. Meanwhile, the ethics committee of Shanghai Ninth People's Hospital waived the need for informed consent (protocol code SH9H2021‐T238‐2).

## Conflicts of Interest

The authors declare no conflicts of interest.

## Supporting information


**Figure S1.** Femoral neck‐shaft angle measurement.

**Figure S2.** Femoral anteversion measurement.
