# Supplementary figures and images for: Positive Correlation Between Acetabular Anteversion and Abduction in Developmental Dysplasia of the Hip: A CT‐Based Morphological Study
Source: Orthop Surg. 2025 Apr 9;17(6):1782–90. doi: 10.1111/os.70037 (PMC12146110; doi:10.1111/os.70037)

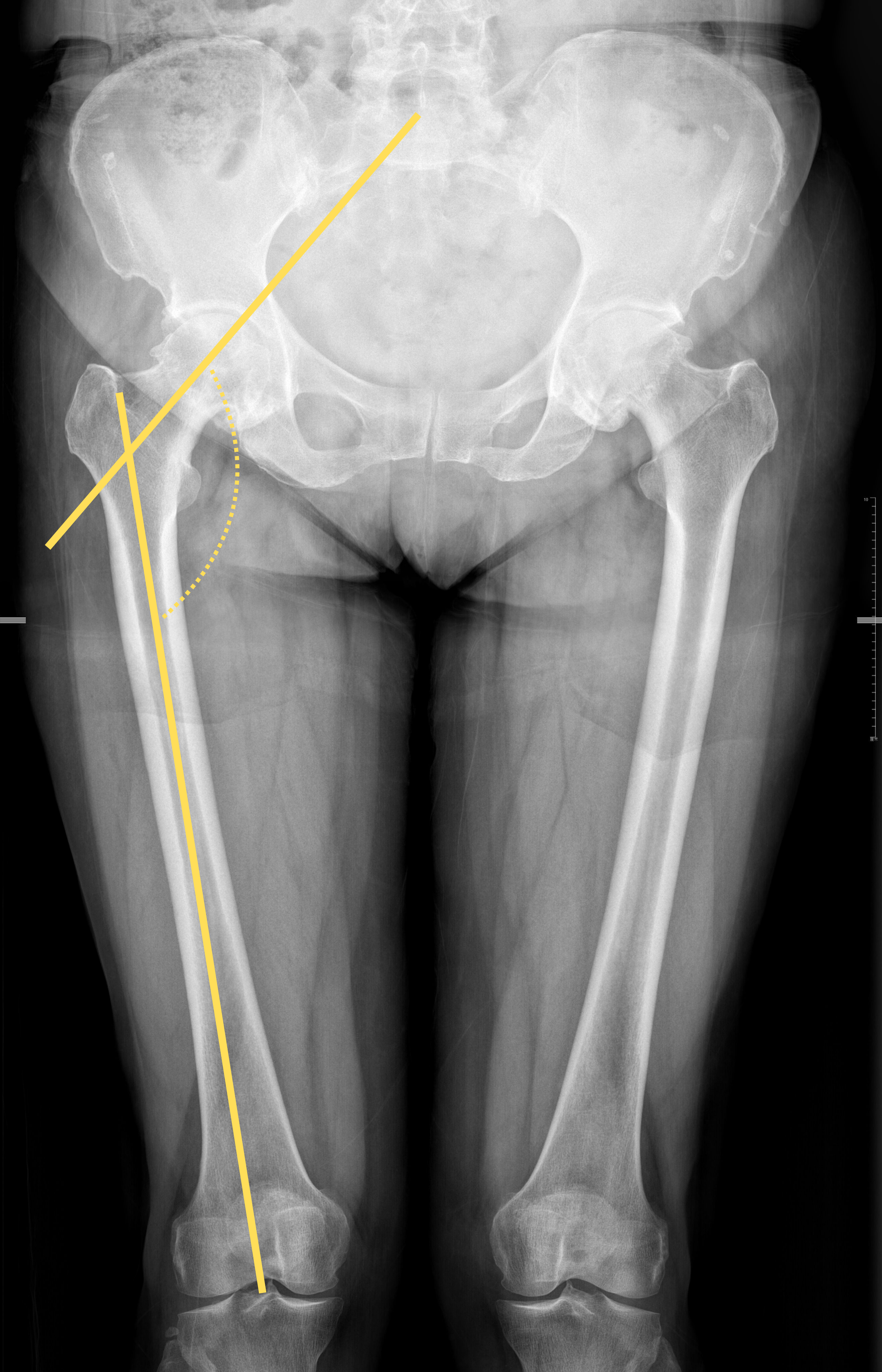

Supplement: Supplementary file 1 — Figure S1. Femoral neck‐shaft angle measurement. [file OS-17-1782-s002.png]

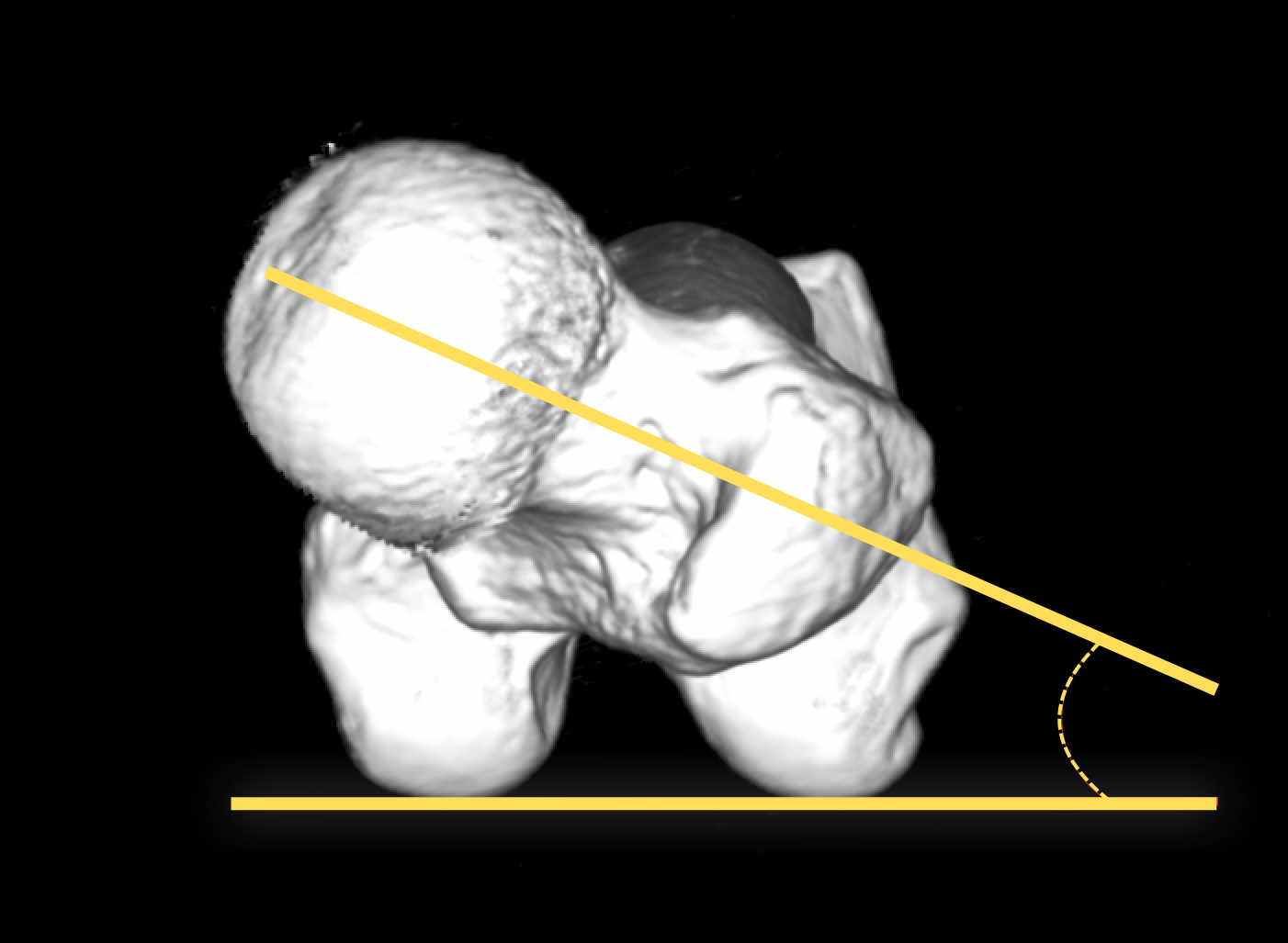

Supplement: Supplementary file 2 — Figure S2. Femoral anteversion measurement. [file OS-17-1782-s001.png]
